# The miR526b-5p-Related Single Nucleotide Polymorphisms, rs72618599, Located in 3'-UTR of *TCF3* Gene, is Associated with the Risk of Breast and Gastric Cancers

**DOI:** 10.52547/ibj.26.1.53

**Published:** 2021-10-27

**Authors:** Maryam Mohammadi, Ali Salehzadeh, Soheila Talesh Sasani, Alireza Tarang

**Affiliations:** 1 Department of Biology, Rasht Branch, Islamic Azad University, Rasht, Iran;; 2 Department of Biology, Faculty of Science, University of Guilan, Rasht, Iran;; 3 Rice Research Institute of Iran, Agricultural Research, Education and Extension Organization (AREEO), Rasht, Iran

**Keywords:** Breast cancer, Gastric cancer, MicroRNA, TCF3

## Abstract

**Introduction:**

Single nucleotide polymorphisms result in dysregulation of the proto-oncogene *TCF3 *gene, which is associated with the development, metastasis, and chemoresistance of different malignancies.

**Methods:**

GSE10810 microarray dataset and GEPIA2 online software were used to find differentially expressed genes and the *TCF3 *status in BC and GC, respectively. Plots and figures of microarray analysis were prepared by ggplot2 and pheatmap packages. Differentially expressed genes were obtained by the Bioconductor limma package. *In silico* analysis was used to predict the functions of rs72618599. BC (n = 123), GC (n = 132) and healthy age and gender matched controls (n = 184) were genotyped, using the high-resolution melting technique.

**Results:**

Based on the allelic comparison study, C allele of rs72618599 was associated with the BC tumor stage IV (66.1%, 78/120, *p* < 0.0001) and grade III (52.4%, 55/72, *p* < 0.0001), while the T allele was associated with metastasis (84.2%, 10/162, *p* < 0.0001). However, in GC patients, the C allele was significantly correlated with *H. pylori *infection (51.7%, 30/58*,* *p* = 0.008), stage III of primary tumors (47.7%, 62/88, p=0.017), stage II of lymph node status (35.5%, 44/74, *p* = 0.017), and metastasis (52.9%, 90/132, *p* = 0.044). *In silico* analysis predicted that rs72618599 leads to the creation of a binding site for hsa-miR526b-5p in the 3′-UTR of *TCF3* transcript.

**Conclusion:**

Regarding the rs72618599 SNP, the C allele, is associated with poor prognosis of BC and GC. Furthermore, rs72618599 may be associated with cancer progression by altering the regulatory affinity of hsa-miR526b-5p to 3′-UTR of *TCF3*.

## INTRODUCTION

Breast cancer is the most prevalent cancer and the second leading cause of cancer-associated death in women worldwide^[^^1^^]^. In recent years, BC has been the most commonly diagnosed cancer among Iranian females with an increased incidence of 16.0-28.3 per 100,000 women^[^^2^^,^^3^^]^. 

GC remains the fourth most frequent malignant neoplasm and the second main reason for cancer mortality with about 800 deaths per 100,000 patients globally^[^^1^^]^. In Iran, GC is a major healthcare problem with approximately 10,000 new cases and 8,000 mortalities per year^[^^4^^]^. This cancer is a multifactorial malignancy affected by the interaction of genetic and environmental factors^[^^5^^]^. It has been reported that the interaction of gastric cells with *H. pylori* and its CagA oncoprotein plays an important role in GC development^[^^6^^]^. However, other environmental factors, including dietary, smoking, gastrointestinal microbiota etc. could be also associated with GC development^[^^6^^]^. 

Due to the increasing mortality and morbidity of BC and GC in the Iranian population, characterization of the prognostic factors such as genetic determinants would be helpful for the disease screening and early treatment approaches. Many researchers have identified that SNPs in some loci can affect tumor susceptibility^[^^7^^,^^8^^]^, progression^[^^8^^,^^9^^]^, and metastasis of BC and GC^[^^10^^,^^11^^]^. Polymorphisms are located in different regions of genes, comprising promoters, coding sequence (introns and exons), and UTR (5′- and 3′)^[^^12^^]^. *TCF3*, also called E2A, is a member of the *TCF* family, which is involved in the regulation of the Wnt signaling pathway and E-cadherin expression^[^^13^^,^^14^^]^. Overexpression of the *TCF3* has been reported in various cancers, including BC and GC^[^^15^^,^^16^^]^. Although numerous SNPs are correlated with the increased risk of BC and GC, no SNP in the *TCF3* has been identified to be associated with GC^[^^7^^-^^11^^]^.

miRNAs are small and regulatory RNAs that bind to the 3′-UTR region of different target mRNAs and contribute to various human malignancies such as GC^[^^17^^]^. MiRNA-526b is located in 19q13.42, and its dysregulation has an important role in the progression of various cancers^[^^18^^]^. This miRNA has been reported to be abnormally expressed in different cancers such as BC and GC^[^^18^^-^^20^^]^. Some SNPs, known as miR-SNPs, affect miRNA binding sites in the 3′-UTRs of target genes and assist in the susceptibility of various types of cancers^[^^8^^]^. MiR-SNPs are functional SNPs that may have an effect on miRNA function^[^^21^^]^. For instance, miR-SNPs in the 5′-UTR regulate the translation initiation of target mRNAs, whereas mRNA stability is determined by SNPs in the 3′-UTR^[^^21^^]^.

As described above, the binding affinity of miRNAs could be affected by SNPs at 3′ UTR of the* TCF3 *gene, which could be associated with the increased risk of different cancers, including BC and GC. Therefore, in the present study, we aimed to elucidate the role of rs72618599 SNP in the susceptibility and development of BC and GC. To our knowledge, the possible association between this SNP and the risk of BC and GC has not yet been studied. Thus, the present study is the first report for the association between rs72618599 with the risk of BC and GC among an Iranian population.

## MATERIAL AND METHODS

**Bioinformatics approaches**

With focusing on high-throughput tests, we conducted a microarray analysis by R studio (4.0.2) software to find the differentially expressed genes in BC and GC. For BC diagnosis, 31 tumor and 27 control tissue samples in GSE10810 dataset were analyzed. The microarray raw data were obtained by GEOquery package (https://bioconductor.org/packages /release/bioc/html/GEOquery.html). Also, differential expression gene analysis was performed by Limma package (https://www.bioconductor.org/packages/ release/bioc/html/limma.html). Normalization of raw data was performed by quantile normalized method. These two packages were obtained from Bioconductor. Based on the distribution of the expression data of the genes studied in this experiment, the genes with logFC greater than the third quartile and logFC smaller than the first quarter were selected as up-regulated and low expressed genes, respectively. Plots and figures of microarray analysis were prepared by ggplot2 and heatmap packages. GEPIA2 online software (http://gepia2.cancer-pku.cn/) was used to find the differentially expressed gene in GC. The GEPIA2 analysis was based on TCGA RNA-seq data. 

**GSEA analysis**

For pathway enrichment analysis, GSEA software (https://www.gsea-msigdb.org) was used to compute the high and low expressed genes in the expression data of microarray analysis and present the relevant signaling pathways to these up-regulated and down-regulated genes.

**Study population**

A total number of 255 patients (123 BC and 132 GC cases) and 184 controls (132 and 52 for BC and GC, respectively) participated in this study. The healthy controls were selected randomly and were age-matched with the cases. The participants were selected from the individuals referring to the Sayed Al Shohada Hospital, Isfahan, Iran. Demographic and clinical characteristics of the subjects, including their blood group, ER, PR, *H. pylori* infection, history of cancer among their relatives etc. were determined by laboratory tests and consent forms. Pathophysiological features of the patients were presented in Table S1. 

**Genotyping by real-time RT-PCR HRM analysis**

**Fig. 1:**
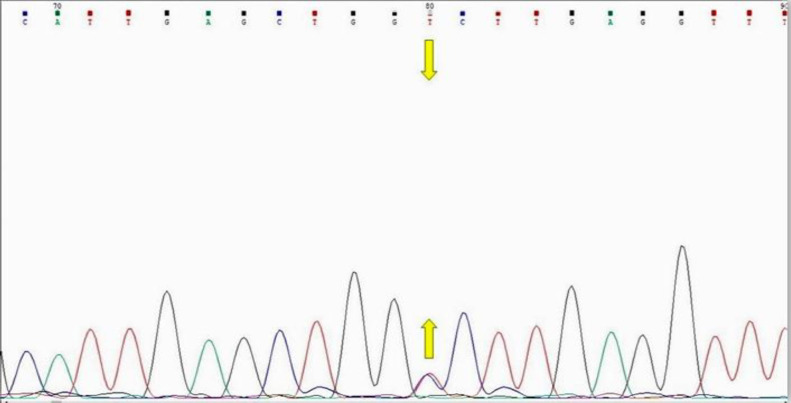
Sequencing chromatogram of rs72618599 heterozygous genotype for *TCF3* gene

Peripheral blood samples (3 mL) were collected from the subjects, and genomic DNA was extracted using PrimePrep genomic DNA isolation kit (GeNet Bio, Korea), according to the *manufacturer’s protocol*. The quality and quantity of the extracted DNA were determined using the NanoDrop™ *spectrophotometry* and 1% agarose gel electrophoresis. Amplification of target region for the rs72618599 was performed using the PCR and HRM methods as described previously, with minor modifications^[^^22^^]^. The reaction mixture contained 2 µL of template DNA, 1 µL of each forward and reverse primer (10 Pico mole), 171.5 µL of PCR master mix, and 5 µL of deionized distilled water. Also, 2 µL of EvaGreen (Solis Biodyne, Estonia) was used as an intercalating dye. The primers used in this study were presented in Table S1. The cycling condition was as follows: pre-incubation for 15 min at 95 °C and then 45 c*ycles of* denaturation for 15 s at 95 °C, annealing for 20 s at 60 °C, and extension for 20 s at 72°C (Table S1). Finally, the amplified fragments were sequenced to determine the genotypes (Pishgam company, Iran; Fig. 1). 

***In silico*** **studies**

The sequence and other information of SNP rs72618599 in the 3′-UTR of *TCF3 *on chromosome 19p13.3 at position 1609761 were determined using NCBI and GenBank databases. Online prediction software mrSNP (http://mrsnp.osu.edu/), PolymiRTS v 3.0 (http://compbio.uthsc.edu/miRSNP/), and miRSNP (http://cmbi.bjmu.edu.cn/mirsnp) were exploited to predict the miRNA with binding ability to the *TCF3* gene 3′-UTR region containing rs72618599^[^^23^^]^. The interaction of miR with the 3′-UTR after allele alteration was identified using miRNASNPV2.0 software (http://www.bioguo.org/miRNASNP2/ geneTargets.php).

**Statistical analysis**

The SPSS version 21.0 and SNP analyzer software were used for statistical analysis of the data. The Shapiro-Wilk and Kolmogorov Smirnov normality tests were exploited to analyze the normality of data distribution. *Chi*-*square test* was used to assess the Hardy-Weinberg equilibrium in patient samples, *and the correlation between *rs72618599 and clinical characteristics were determined using Pearson’s Chi-square test. Furthermore, the *p *value ˂0.05 was *considered statistically significant*.

**Ethical statement**

The above-mentioned sampling was approved by the Ethical Committee of Islamic Azad University of Rasht, Iran (ethical code: IR.IAU.RASHT.REC. 1398.056). Also, the informed consents were obtained from the subjects before participation in this study. 

## RESULTS

**Bioinformatics analyses**

**Fig. 2:**
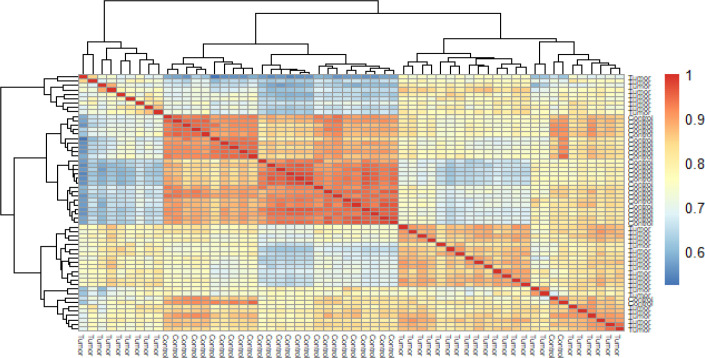
Heatmap of correlation among the samples of GSE10810 dataset. The Figure shows the high quality of the samples because both the control samples and tumor samples are correlated

**Fig. 3:**
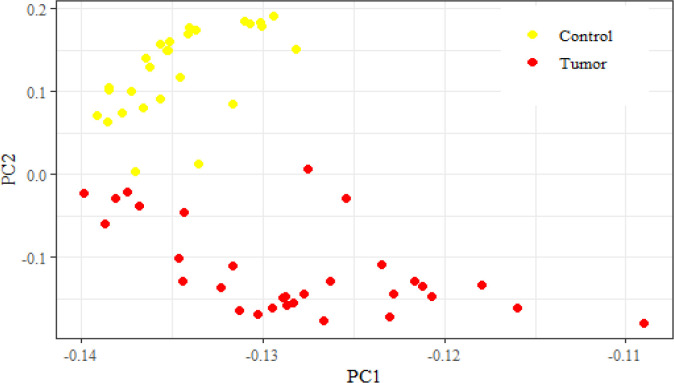
Principal component analysis plot of GSE10810 dataset, indicating a remarkable difference between the normal and tumor samples in expression pattern of genes

**Fig. 4.**
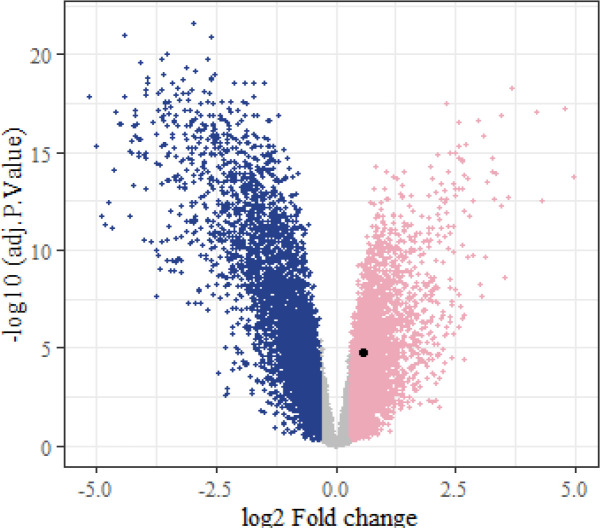
Volcano plot showing the differentially expressed genes in the GSE10810 dataset. This plot was drawn by the logFC and -log10 (adj *p*) of genes. Up-regulation of TCF3 is demonstrated by the black point

**Fig. 5:**
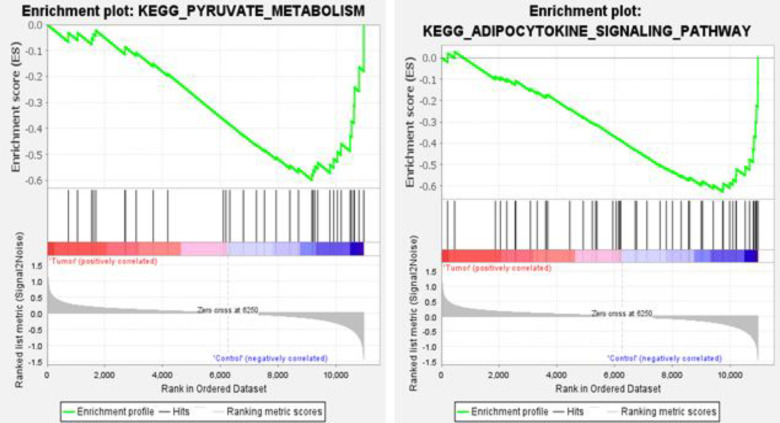
GSEA analysis, suggesting the involvement of differentially expressed genes in pyruvate metabolism and adipocytokine signaling pathways

Microarray analysis on GSE10810 dataset revealed that this dataset had 4596 up-regulated and 4596 down-regulated genes in tumor samples as compared to the normal tissue (Figs. 2-4). Based on adj *p*, the *TCF3* was significantly up-regulated in this dataset (adj *p*= 1.762595e-05; logFC = 0.56). According to GEPIA2 online software result, *TCF3* had a significantly increased expression in GC samples, as compared to the normals (adj *p* = 9.72e-63; logFC = 1.508). GSEA pathway enrichment analysis revealed that the up-regulated genes of GSE10810 microarray expression data are involved in the pyruvate metabolism and adipocytokine signaling pathways (Fig. 5). 

**Frequency and association of rs72618599 in human BC**

A total number of 255 participates, including 123 BC patients (mean age of 52.88 ± 11.98 years) and 132 controls (mean age of 17 to 73 years) were studied to determine the association between rs72618599 and the BC risk. Table S2 summarizes the clinical characteristics of the BC cohort. Results showed that the rs72618599 was not in the Hardy-Weinberg 

**Frequency and association of rs72618599 in human GC**

 Clinical characteristics of the GC cohort (130 patients and 54 controls) are listed in Table S3. The controls were age-matched with the cases and selected from people without any history of cancer. The SNP analyzer software (SNPanalyzer v2.0) revealed that rs72618599 was not in the Hardy-Weinberg equilibrium (*p *= 0.000). The association of different rs72618599 genotypes with GC risk was studied (Table 1). The statistical analysis showed that the CC and CT genotypes were more common among GC patients (54.5% and 30.3%, respectively) than controls (53.9% and 19.2%, respectively). However, no significant relationship was found between rs72618599 genotypes and GC patients (*p *˃ 0.05). Based on the results (Table 3), the majority of GC patients were A+ blood type (39.66%), and the CC genotype was the most prevalent genotype in 24 out of 46 patients. Also, the lymph node status of the patients was significantly associated with the rs72618599 genotypes. We found that the CC and CT genotypes were associated with lymph node status III among GC patients. In addition, no significant relationship with smoking, *H. pylori *infection, primary tumor status, cancer stage, and metastasis was found for rs72618599 genotypes (Table 3). Evaluating the allele frequency revealed that the C allele was significantly associated with *H. pylori *infection (51.7%, *p* = 0.008), primary tumor status (47.7% for the third stage, *p* = 0.017), lymph node status (35.5%; for the second stage; *p* = 0.017), and positive metastasis (52.9%; *p = *0.044). There was no direct correlation between the allele frequency and blood group, smoking, and cancer stage (Table 3).

***In silico*** **analysis**

**Fig. 6:**
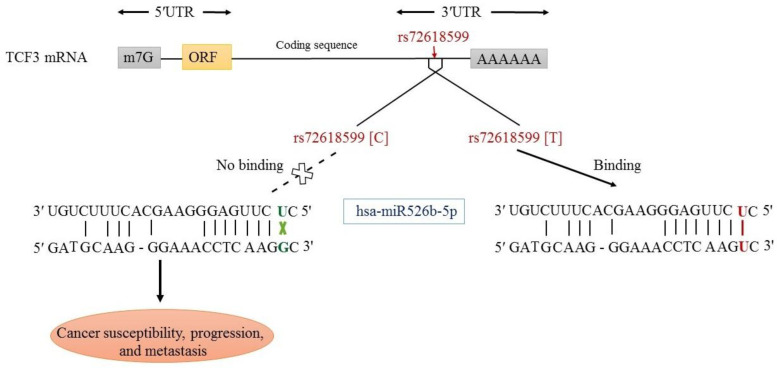
The proposed functional effect of rs72618599 on hsa-miR526b-5p binding to *TCF3 *3′-UTR

As rs72618599 is located in the 3′-UTR of the *TCF3* gene, we postulated that this variant may impose its effect on altering the interaction of *TCF3* mRNA with miRNAs. Using online bioinformatics software miRBase (http://www.mirbase.org) and miRNASNP V2.0, it was identified that T allele can alter the binding potential of hsa-miR526b-5p. The substitution of the C allele at rs72618599 for the T allele can produce an illegitimate canonical hsa-miR526b-5p recognition site in the *TCF3 *gene (Fig. 6). 

**Table 1:** Genotype and allele frequency of rs72618599 polymorphism among the patients (BC and GC) and healthy controls

**Cancer type**	**Groups**	**Genotype frequency n (%)**				**Allele frequency n (%)**	
		**CC**	**CT**	**TT**		**C**	**T**
BC	Cases	48 (39.0)	30 (24.4)	45 (36.6)		126 (51.2)	120 (48.8)
	Controls	63 (47.7)	27 (20.5)	42 (31.8)		153 (58.0)	111 (42.0)
		χ^2 ^= 1.97, *p* = 0.373				χ^2 ^= 2.33, *p* = 0.126	
							
GC	Cases	72 (54.5)	40 (30.3)	20 (15.2)		184 (69.7)	80 (30.3)
	Controls	28 (53.9)	10 (19.2)	14 (26.9)		66 (63.5)	38 (36.5)
		χ^2 ^= 4.48, *p* = 0.106				χ^2 ^= 2.33, *p *= 0.126	

## DISCUSSION

TCF3 is a transcriptional repressor associated with the initiation and growth development of tumors^[^^24^^]^. Previous researches have indicated an association between the overexpression of *TCF3* and different cancers, including breast, colorectal, cervical, prostate cancers, and GC^[^^15^^,^^25^^]^. MiRNAs have been introduced as *TCF3* regulatory molecules because of their binding to the 3′-UTR region of the gene transcript, thus regulating the expression of the target gene^[^^15^^,^^25^^]^. SNPs in the binding site of miRNAs could affect binding affinity to the target transcripts and, thus, interrupts the regulatory functions of miRNAs which in turn may translate to cancer initiation^[^^15^^,^^24^^,^^25^^]^. 

The association of the SNPs at 3′-UTR region of *TCF3* gene with BC and GC risk has been rarely investigated. For the first time in this work, we evaluated the correlation of 3′-UTR rs72618599 SNP of *TCF3* gene with BC and GC among an Iranian population and predicted its outcomes by bioinformatics and *in silico* analyses. Evaluating the association of the 3′-UTR rs72618599 SNP with BC risk showed that this SNP was not significantly associated with the increased cancer risk. However, a significant association was observed between the CC genotype and tumor grading. Also, the tumor metastasis was mainly associated with the CT and TT genotypes. These findings disclose that several genetic factors could be associated with tumor initiation, development, and metastasis in BC. In fact, the miR-SNPs in the 3′UTRs of a gene not only can affect miRNAs binding efficiency but also can alter the polyadenylation of the transcript and their interactions with proteins, which significantly affects mRNA stability and their translation regulation^[26]^. Moreover, we found that the 3′-UTR was not associated with the hormonal receptor status of breast tumors. Thus, the rs72618599 SNP was not a major determinant in the development of breast tumors through regulating the expression of hormonal receptors on BC cells. 

**Table 2:** Genotype and allele frequency of rs72618599 SNP in relation to clinical features of BC patients

**Variable**	**No. of genotype n (%)**					**No. of allele (%)**			
	**Total (%)**	**CC**	**CT**	**TT**	***p *** **value**	**Total (%)**	**C**	**T**	***p *** **value**
HER2 receptor					0.745				0.364
HER2(+)	22 (27.16)	6 (23.1)	5 (25.0)	11 (31.4)		44 (27.16)	17 (23.6)	27 (30.0)	
HER2(+)	59 (72.84)	20 (76.9)	15 (75.0)	24 (68.6)		118 (72.84)	55 (76.4)	63 (70.0)	
Estrogen receptor					0.208				0.112
ER(+)	66 (81.48)	21 (77.8)	16 (72.7)	29 (90.6)		132 (81.48)	58 (76.3)	74 (86.0)	
ER(-)	15 (18.52)	6 (22.2)	6 (27.3)	3 (9.4)		30 (18.52)	18 (23.7)	12 (14.0)	
Progesterone receptor					0.438				0.123
PR(+)	60 (74.07)	18 (66.7)	16 (72.7)	26 (81.2)		120 (74.07)	52 (68.4)	68 (79.1)	
PR(-)	21 (25.93)	9 (33.3)	6 (27.3)	6 (18.8)		42 (25.93)	24 (31.6)	18 (20.9)	
Tumor stage					0.007^a^				0.000^a^
I	21 (18.42)	3 (6.7)	6 (21.4)	12 (29.3)		42 (18.42)	12 (10.2)	30 (27.3)	
II	18 (15.79)	6 (13.3)	3 (10.7)	9 (22.0)		36 (15.79)	15 (12.7)	21 (19.1)	
III	15 (13.16)	6 (13.3)	1 (3.6)	8 (19.4)		30 (13.16)	13 (11.0)	17 (15.4)	
IV	60 (52.63)	30 (66.7)	18 (64.3)	12 (29.3)		120 (52.63)	78 (66.1)	42 (38.2)	
Tumor grade					0.001^a^				0.000^a^
I	18 (18.18)	3 (7.7)	5 (18.5)	10 (30.3)		36 (18.18)	11 (10.5)	25 (26.9)	
II	45 (45.45)	12 (30.8)	15 (55.6)	18 (54.5)		90 (45.46)	39 (37.1)	51 (54.8)	
III	36 (36.37)	24 (61.5)	7 (25.9)	5 (15.2)		72 (36.36)	55 (52.4)	17 (18.3)	
Metastasis					0.000^a^				0.000^a^
Positive	81 (65.85)	18 (37.5)	25 (83.3)	38 (84.4)		162 (65.85)	61 (48.4)	101 (84.2)	
Negative	42 (34.15)	30 (62.5)	5 (16.7)	7 (15.6)		84 (34.15)	65 (51.6)	19 (15.8)	

^a^ *p* < 0.05

**Table 3:** Genotype and allele frequency of rs72618599 SNP in relation to clinical features of GC patients

**Variable**		**No. of genotype (%)**				**No. of allele (%)**			
	**Total (%)**	**CC**	**CT**	**TT**	***p *** **value**	**Total (%)**	**C**	**T**	***p *** **value**
Blood group					0.026^a^				0.554
A+	46 (39.66)	24 (40.0)	20 (50.0)	2 (12.5)		92 (39.66)	68 (42.5)	24 (33.3)	
B+	20 (17.24)	12 (20.0)	4 (10.0)	4 (25.0)		40 (17.24)	28(17.5)	12 (16.7)	
B-	2 (1.73)	0 (0)	2 (5.0)	0 (0)		4 (1.72)	2 (1.2)	2 (2.8)	
O+	30 (25.86)	14 (23.3)	12 (30.0)	4 (25.0)		60 (25.86)	40 (25.0)	20 (27.8)	
O-	6 (5.17)	2 (3.3)	2 (5.0)	2 (12.5)		12 (5.18)	6 (3.8)	6 (8.3)	
AB	12 (10.34)	8 (13.4)	0 (0)	4 (25.0)		24 (10.34)	16 (10.0)	8 (11.1)	
									
Smoking					0.364				0.314
Positive	40 (60.6)	22 (68.8)	12 (50.0)	6 (60.0)		80 (60.6)	56 (63.6)	24 (54.5)	
Negative	26 (39.4)	10 (31.2)	12 (50.0)	4 (40.0)		52 (39.4)	32 (36.4)	20 (45.5)	
									
*H.pylori*					0.382				0.008^a^
Positive	29 (53.7)	11 (37.9)	8 (27.6)	10 (34.5)		58 (59.79)	30 (51.7)	28 (48.3)	
Negative	25 (46.3)	11 (44.0)	12 (48.0)	2 (8.0)		39 (40.21)	23 (59.0)	16 (41.0)	
									
Primary tumor status					0.113				0.017^a^
I	2 (2.17)	0 (0)	0 (0)	2 (12.5)		4 (2.17)	0 (0)	4 (7.4)	
II	32 (34.78)	20 (37.0)	8 (36.3)	4 (25.0)		64 (34.78)	48 (36.9)	16 (29.6)	
III	44 (47.83)	26 (48.2)	10(45.5)	8 (50.0)		88 (47.83)	62 (47.7)	26 (48.2)	
IV	14 (15.22)	8 (14.8)	4 (18.2)	2 (12.5)		28 (15.22)	20 (15.4)	8 (14.8)	
									
Lymph node status					0.023^a^				0.017^a^
0	16 (17.77)	12 (24.0)	0 (0)	4 (25.0)		32 (16.84)	24 (19.4)	8 (12.1)	
I	32 (35.56)	18 (36.0)	8 (33.3)	6 (37.5)		74 (38.95)	44 (35.5)	30 (45.5)	
II	24 (26.67)	8 (16.0)	10 (41.7)	6 (37.5)		48 (25.26)	26 (21.0)	22 (33.3)	
III	18 (20)	12 (24.0)	6 (25.0)	0 (0)		36 (18.95)	30 (24.1)	6 (9.1)	
									
Tumor stage					0.150				0.105
I	14 (11.86)	8 (11.8)	2 (6.2)	4 (22.2)		28 (11.67)	18 (10.7)	10 (13.9)	
II	24 (20.34)	14 (20.6)	6 (18.8)	4 (22.2)		48 (20)	34 (20.2)	14 (19.4)	
III	36 (30.51)	16 (23.5)	12 (37.5)	8 (44.4)		72 (30)	44 (26.2)	28 (38.9)	
IV	44 (37.29)	30 (44.1)	12 (37.5)	2 (11.2)		92 (38.33)	72 (42.9)	20 (27.8)	
									
Metastasis					0.067				0.044^a^
Positive	66 (57)	36 (52.9)	18 (52.9)	12 (85.7)		132 (56.9)	90 (52.9)	42 (67.7)	
Negative	50 (43)	32 (47.1)	16 (47.1)	2 (14.3)		100 (43.1)	80 (47.1)	20 (32.3)	

^a^ *p* < 0.05

 We also explored that the C allele of rs72618599 was significantly associated with the increased risk of stage IV of tumor and tumor grade III, indicating its role in the severity of breast tumors, while the presence of the T allele was mainly contributed to the increased risk of cancer metastasis. Similarly, we observed no association between rs72618599 genotypes and GC risk. Many studies have reported the effect of SNPs at 3′-UTR of a variety of genes on the binding efficacy of miRNAs and their association with different cancers^[^^24^^,^^27^^,^^28^^]^. Unlike the majority of reports, the present study found no association of the rs72618599 SNP with BC and GC.

TCF3 plays a fundamental role in tumor initiation and growth. This protein works as a transcriptional regulator with the Wnt signaling pathway^[^^24^^]^. In fact, Wnt/β-catenin signaling is related to the activation of many genes via the interaction of TCF3 with the β-catenin^[^^24^^]^. Thus, the efficient silencing of the TCF3 by RNAi could inhibit the activation of the genes involved in cell proliferation. Previous works have suggested that silencing of TCF3 by RNAi causes cell cycle arrest at the G2 phase and represses the proliferation of cancer cells^[^^24^^,^^29^^]^. In a previous study, Kumar *et al.*^[^^30^^]^ reported that the reduction of TCF3 level decreased tumor formation and reduced tumor growth rate. Similarly, it was reported that the silencing of TCF3 gene can led to the growth, proliferation, and colony formation of GC cells^[^^31^^]^. Furthermore, downregulation of TCF3 was associated with the reduction of *Bcl2* and increased expression of the *Bax* gene, which results in apoptosis induction^[^^24^^]^. Thus, the reduction of *TCF3* expression by miRNAs has a critical role in the inhibition of tumor initiation and development. In this work, we observed no association of the rs72618599 SNP at 3′-UTR of the *TCF3* gene with BC and GC. Hence, it seems that the binding efficacy of miRNAs to the 3′-UTR of the *TCF3* gene is not the only determinant in breast and gastric tumor development. Also, some alleles were associated with tumor grade and their pathological characteristics. Further experiments are required to elucidate the effect of this polymorphism on the pathological features of breast and gastric tumors.

Microarray analysis showed that TCF3 had a slight and insignificant up-regulation (LogFC = 0.26, adj *p *= 0.33) in GC. It was also significantly up-regulated in BC (LogFC = 0.4, adj *p* = 0.000). The has-miR-526-5p was down-regulated slightly (LogFC = -0.29, adj *p *= 0.35) in tumor samples compared with normal samples. GSEA analysis exhibited that the genes involved in pyruvate metabolism and adipocytokine signaling pathways significantly reduced. Our work revealed that the rs72618599 T allele might alter the binding potential of hsa-miR526b-5p to the 3′-UTR of the *TCF3* gene. The stability of the interaction between hsa-miR526b-5p and TCF3 mRNA declined for the C allele. Therefore, the *TCF3 *gene is probably overexpressed in these patients and leads to the development of BC and GC.

In this case-control study, the contribution of rs72618599 SNP in *TCF3 *with the risk of BC and GC among the Iranian population was investigated for the first time. Our results revealed no significant association of the SNP with BC and GC risk. However, the C allele could be involved in tumor development and severity and the T allele could be contributed to tumor metastasis. Our findings also demonstrated that the T allele may alter the binding affinity of hsa-miR526b-5p to the 3′-UTR region of the *TCF3* gene, which may result in a higher expression of this transcription factor.

## CONFLICT OF INTEREST

None declared.
